# Could Weaning Remodel the Oral Microbiota Composition in Donkeys? An Exploratory Study

**DOI:** 10.3390/ani12162024

**Published:** 2022-08-10

**Authors:** Zhenwei Zhang, Bingjian Huang, Yonghui Wang, Mingxia Zhu, Changfa Wang

**Affiliations:** Liaocheng Research Institute of Donkey High-Efficiency Breeding and Ecological Feeding, Agricultural Science and Engineering School, Liaocheng University, Liaocheng 252059, China

**Keywords:** oral microbiome, weaning, 16S rRNA, microbial function, donkey foal

## Abstract

**Simple Summary:**

Weaning is a stressful event as it is an abrupt separation of donkey foals from their mothers. Donkeys experience a variety of changes and stresses, such as physiological, environmental and diet challenges. The oral cavity is the gateway to total body wellness, and the oral microbiome is important in maintaining oral as well as systemic health. However, little is known on the effect of weaning on the oral microbiome of donkeys. Therefore, the present study was conducted to investigate the relationship between weaning and oral microbial changes using 16S rRNA gene sequencing. The results showed that significant changes in the oral microbiome occurred during the weaning transition period. In addition, the functions related to carbohydrate metabolic pathways were obviously enriched in the oral microbiome in postweaning donkeys compared with preweaning donkeys. The current study provides a deeper insight into the oral microbiota changes during the weaning period and helps to increase awareness of feed changes in donkey foals.

**Abstract:**

As the initiation point of digestion, the oral microbiome is important in maintaining oral and systemic health. However, the composition of oral microbial communities and the influence of weaning on the oral microbiota of donkey foals remains poorly characterized. The present study used buccal swab samples to determine the changes in oral microbial communities occurring at the time of weaning. A total of 20 oral swab samples were collected from two groups: preweaning donkey foals (PreW group, *n* = 10) and postweaning donkey foals (PostW group, *n* = 10). The donkey oral microbiome was analyzed by 16S rRNA gene sequencing using Illumina MiSeq. This study is the first report of the donkey oral microbiome in association with weaning. Compared to the preweaning donkeys, the oral bacteria diversity in the postweaning donkeys was increased, with a higher Simpson index. Changes in the composition of the oral microbiota between the PreW and PostW groups were observed in the present study. At the phylum level, the relative abundance of Firmicutes and Myxococcota was significantly greater in the PostW than in the PreW group. At the genus level, the *Gemella*, unclassified_o__*Lactobacillales*, and *Lactobacillus* were increased in the postweaning donkeys. The donkeys’ oral microbial functions were predicted using PICRUSt, and the functions related to carbohydrate metabolic pathways were significantly enriched in the oral microbiome in the PostW donkeys. In summary, the current study provides a deeper insight into the oral microbiota changes during the weaning period, and the influence of weaning together with the documented changes in diversity and composition will help us to obtain a better understanding of their long-term health impact within the oral cavities of donkey foals. However, a major limitation of the present study was that the samples were obtained from different animals in the PreW and PostW groups, which may have resulted in variability among the different individuals. Further investigation is needed to monitor the shift in oral microbes in the same individuals during the weaning period.

## 1. Introduction

In recent years, donkey farming has gained popularity in several countries, where these equids are mainly reared for milk production [1,2]. Thanks to its special properties, donkey milk is suitable for infants who cannot be breast-fed and people suffering from cow’s-milk-protein allergies [3]. Donkeys reared for milk production need to be continuously managed and monitored to maintain optimal general health conditions, avoiding nutritional deficiencies [4]. Several researches have focused their attention on the physiology of this equid species [5,6]; however, more detailed information is required in order to optimize in animal health and wellbeing.

The donkey is a typically hindgut fermenter that relies on a high input of fibrous feedstuffs, which are degraded and fermented to produce energy for the host [7]. The donkey’s digestive physiology is characterized by a long and intense microbial fermentation in the hindgut. Recently, the donkey hindgut microbiota has been intensively investigated [8,9] and it is well known to perform an important role in the animal’s health and well-being [10,11].

However, the overall digestive system of donkeys is a hollow tube that starts with the oral cavity. The oral cavity is comprised of many surfaces, each coated with a plethora of bacteria, fungi, and protozoa [12]. Bacterial oral colonizers may be crucial to the initial establishment of the complex gut microbial community [13]. Currently, little is known regarding the microbiota of the healthy oral cavity in donkeys.

Weaning is a stressful event as it is an abrupt separation of young animals from their mothers. During the weaning transition, donkeys experience a variety of changes and stresses, such as physiological, environmental, and social challenges [13]. In particular, the diet of donkeys abruptly shifts from a high-fat, low-fiber milk to a high-fiber and low-fat solid feed. It has been shown that the weaning process and the introduction of plant-based solid feed are important driving forces in the succession of gut bacteria in animals [14]. In the previous study, Mach et al. [15] reported that the maternal separation at weaning remodeled the gut microbiota of foals during the first day post-weaning. In addition, the rumen bacterial communities changed dynamically throughout the weaning period in calves [16]. However, the oral cavity is the primary partition exposed to the diet shifts during the weaning process. There is limited information available regarding the structure and function of the oral microbiome of donkeys within the weaning transition.

In addition to being the initiation point of digestion, the oral microbiome plays an important role in maintaining oral and systemic health [6]. Due to the high prevalence and adverse influence of dental diseases in equines, available studies in horses and donkeys have focused on the oral microbiome and its association with periodontal diseases [17,18]. Although the establishment of the gut microbiota composition has been demonstrated to be affected by the cessation of breastfeeding and weaning onto feedstuffs [19,20,21,22,23], it remains unclear whether the weaning transition influences the oral microbiota composition with the adaptation to new food in donkeys. Therefore, the present study was conducted to explore the impact of weaning on the oral microbiota in donkeys and characterize the primary changes in its composition and functions.

## 2. Materials and Methods

### 2.1. Animals and Sample Collection

The donkeys enrolled in the present study were of the Dezhou breed from a provincial Dezhou donkey original breeding farm authorized by Shandong Province, in China. A total of 20 male donkey foals were selected randomly. Before weaning, the donkey foals were housed together with their mares in a large pen with sand bedding. The foals received donkey milk and fresh water ad libitum. The donkey foals on this farm were weaned at 21 weeks of age (days 147). After weaning, all foals were separated from their mothers and housed together into one sand-bedded barn under same housing conditions. They were fed ad libitum with wheat straw and a commercial concentrate diet (Hekangyuan Co., Ltd., Dezhou, Shandong Province, China). All donkey foals were clinically healthy and examined with parasite screening. During the experimental period, none of the selected foals had signs of enteric or metabolic disturbances from birth until sampling.

Before morning feeding, between 08:00 and 10:00 am on the same day, oral samples were collected from two groups: the preweaning donkey foals (PreW group, *n* = 10) and the postweaning donkey foals (PostW group, *n* = 10). Buccal swabs were collected by using two sterile cotton wool swabs (BKMAM Biotechnology Co., Ltd., Changde, Hunan Province, China) rubbed around the buccal side bilaterally in the mouth of each foal for at least 30 s [18], at 1 week just prior to weaning and after weaning for PreW and PostW group, respectively. Each swab was then immediately inserted into a sterile tube and stored in ice until deep-freezing at −70 °C while awaiting analysis.

### 2.2. The DNA Extraction

According to the manufacturer’s instructions, genomic DNA of the oral swabs was extracted using the Hi-Swab DNA Kit (Tiangen, Beijing, China). The DNA concentration and purity were measured by a NanoDrop 2000 UV-vis spectrophotometer (Thermo Scientific, Wilmington, NC, USA) and 1% agarose-gel electrophoresis.

### 2.3. The PCR Amplification

The V3–V4 hyper-variable regions of the ribosomal 16S rRNA gene were amplified by the ABI GeneAmp^®^ 9700 PCR thermocycler (ABI, Foster City, CA, USA) using the specific primers 338F (5′-3′ ACTC CTAC GGGA GGCA GCAG) and 806R (5′-3′ GGAC TACH VGGG TWTC TAAT) with 10-nanogram template DNA. Each PCR was performed in total volume of 20 μL with 2 μL of 10 × TaKaRa rTaq buffer, 2 μL of 2.5 mM dNTPs, 0.8 μL of forward and reverse primers, 0.2 μL of TrTaq polymerase, 10 ng template DNA and ultrapure water. The thermal cycling was carried out with initial denaturation at 95 °C for 3 min, 27 cycles of denaturing at 95 °C for 30 s, annealing at 55 °C for 30 s, extension at 72 °C for 45 s, and one final cycle of 72 °C for 10 min [9]. The resulting PCR product was monitored by electrophoresis on a 2% agarose gel and then purified using the AxyPrep DNA Gel Extraction Kit (Axygen Biosciences, Union City, CA, USA).

### 2.4. The MiSeq 16S rRNA Sequencing

The purified PCR amplicons were pooled in equimolar and paired-end sequenced (300 bp × 2) by Illumina MiSeq system (Illumina, San Diego, CA, USA) at Shanghai Majorbio Bio-pharm Technology Co., Ltd. (Shanghai, China) The raw reads were uploaded to the NCBI Sequence Read Archive (SRA) database (PRJNA851537).

### 2.5. Sequence Data Processing and Analysis

The sequence raw data were quality-filtered by Trimmomatic and merged by FLASH [15]. Operational taxonomic units (OTUs) were clustered with a 97% similarity cutoff using Uparse (version 11, Tiburon, CA, USA). The ribosomal database project (RDP Classifier, Version 2.13) classifier was applied to annotate the bacterial taxa for each representative sequence with a confidence threshold of 70% against the Silva (Version 138) 16S rRNA database. The relative abundance of bacteria was expressed as a percentage (%), and an extended-error bar plot was created to visualize the significant differences between the two groups using the Statistical Analysis of Metagenomic Profiles (STAMP). Alpha diversity indices were determined using the MOTHUR (Version 1.30.2, Ann Arbor, MI, USA), with the following indices: Sobs index, Chao index, Shannon index, and Simpson index. The rarefaction using the transform-count method was applied to the dataset before the beta diversity calculations. A Venn diagram showed the number of OTUs shared between the groups. The beta diversity was calculated by calculating the Bray–Curtis distance using QIIME (Version 1.9.1, Seattle, AZ, USA) and visualized by principal component analysis (PCA) plots. A non-metric multi-dimensional scaling (NMDS) on Bray–Curtis distance was carried out to show the differential abundance of main bacterial genera between groups. The linear discriminant analysis effect size (LEfSe) pipeline was applied to identify the most differentially abundant taxa between the distinct groups with LDA >3.5. The phylogenetic investigation of communities by reconstruction of unobserved states (PICRUSt, Version 1.1.0, Boston, MA, USA) was applied to predict the functional genes of bacteria. PICRUSt transforms OTUs picked up against the Silva database (Version 138) into a taxonomic profile of COG (Clusters of Orthologous Groups) proteins and KEGG (Kyoto Encyclopedia of Genes and Genomes) organisms.

### 2.6. Statistical Analysis

The sequencing data were mainly analyzed on the Majorbio Cloud Platform (https://www.majorbio.com, accessed on 21 February 2022). The differential abundance of bacterial composition at phylum- and genus-level, the alpha-diversity index, the enriched KEGG module, and metabolic pathways and COG functions were compared between the PreW and PostW groups using Wilcoxon rank-sum test in R (Version 4.0.3, Seattle, WA, USA). Significant differences were declared at *p <* 0.05.

## 3. Results

### 3.1. Alpha Diversity

**Figure 1:**
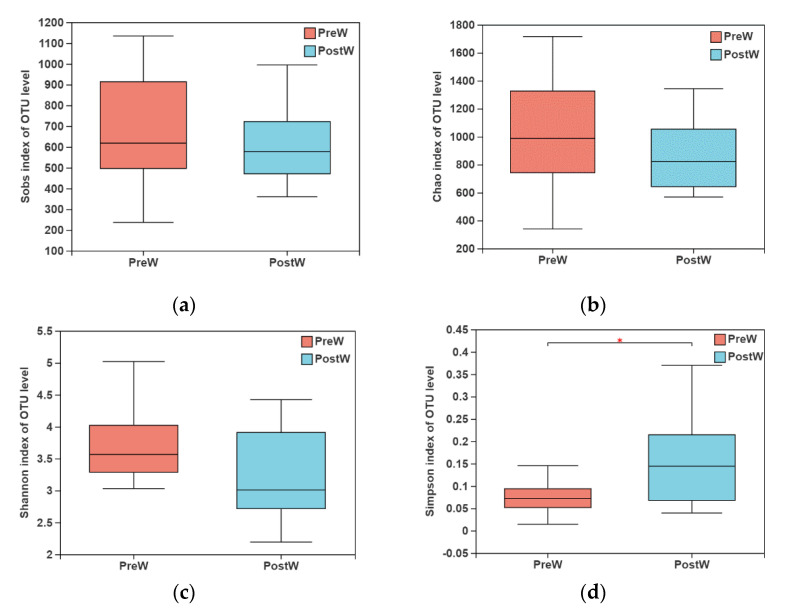
Alpha diversity indices of oral bacteria between preweaning and postweaning donkeys. (**a**) Sobs index; (**b**) Chao index; (**c**) Shannon index; (**d**) Simpson index; PreW, preweaning group; PostW, postweaning group; *, *p <* 0.05.

The microbial richness and diversity were evaluated with α-diversity indices, including the Sobs, Chao, Shannon and Simpson index (Figure 1). There was no significant difference in Sobs, Chao, or Shannon index between the two groups (*p* > 0.05). However, the Simpson index of the oral bacteria in postweaning donkeys was higher than that in preweaning donkeys (*p <* 0.05).

### 3.2. Microbial Composition

**Figure 2:**
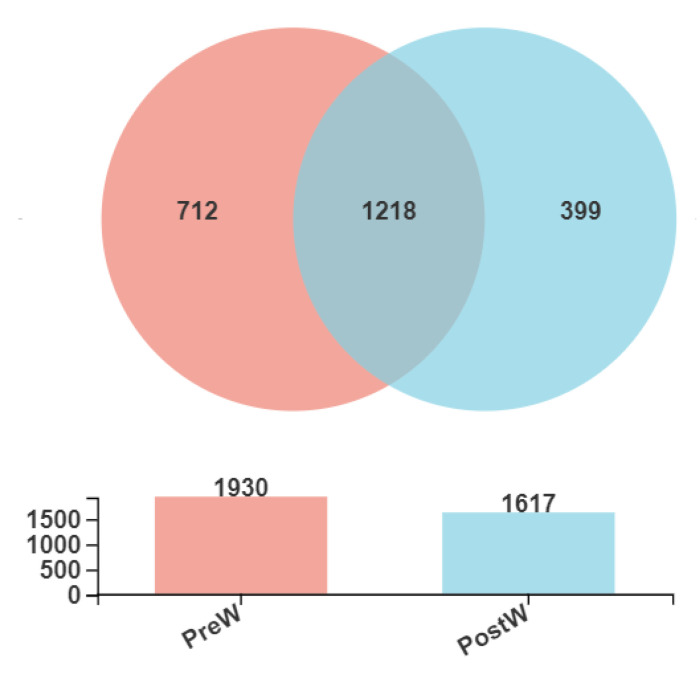
Venn diagram presenting the distribution of oral bacteria community OTUs between preweaning and postweaning donkeys. PreW, preweaning group; PostW, postweaning group.

The Venn diagram in Figure 2 presents the distribution of bacterial community OTUs. In total, 1930 and 1617 OTUs were observed for the oral bacteria in the preweaning and postweaning donkeys, respectively. In addition, the preweaning group shared the oral bacteria community, including 1218 OTUs, with the postweaning group.

**Figure 3:**
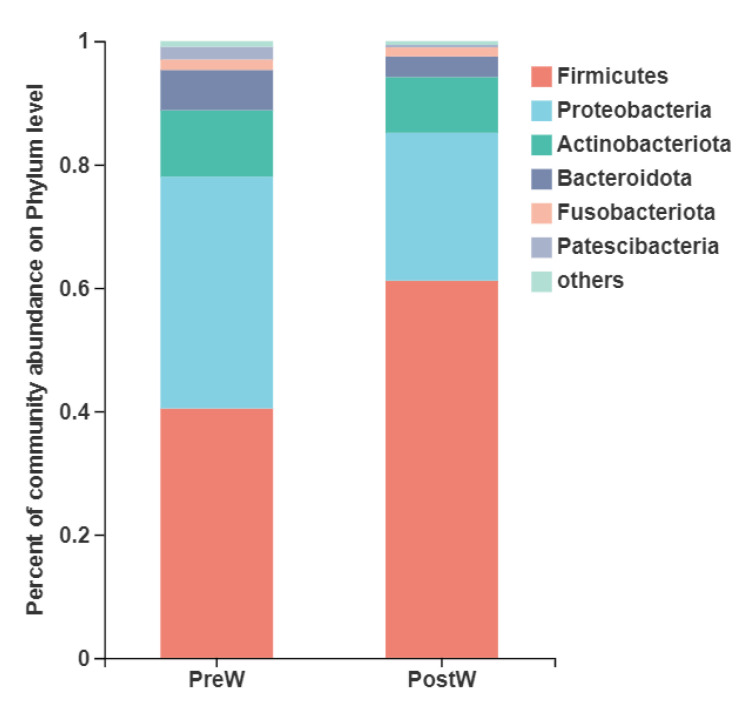
Composition of the predominant oral bacteria at phylum level between preweaning and postweaning donkeys (abundance of the phylum is expressed as %). PreW, preweaning group; PostW, postweaning group.

The oral bacteria with a relative abundance of more than 1% of the total sequences in at least one of the samples were further analyzed (Figure 3). The six predominant phyla were *Firmicutes* (40.4~61.1% of the total sequence reads), *Proteobacteria* (24.0~37.6%), *Actinobacteriota* (9.0~10.8%), *Bacteroidota* (3.3~6.5%), *Fusobacteriota* (1.5~1.7%), and *Patescibacteria* (0.5~2.1%).

**Figure 4:**
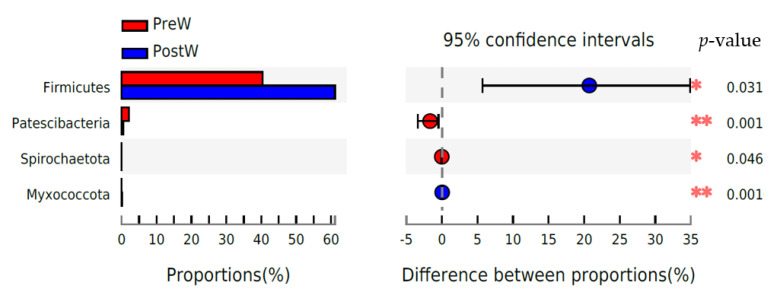
Difference in the predominant oral bacteria at phylum level between preweaning and postweaning donkeys (abundance of the phylum is expressed as %). Extended error bar plot was created by bioinformatics software (STAMP). Welch’s two-sided test was used and Welch’s inverted was 0.95; PreW, preweaning group; PostW, postweaning group. *, *p* < 0.05; **, *p* < 0.01.

The relative abundance of *Firmicutes* and *Myxococcota* was significantly greater in the PostW compared with the PreW group (Figure 4; *p <* 0.05). However, the relative abundance of *Patescibacteria* and *Spirochaetota* was significantly lower in the PostW than that in the PreW group (*p <* 0.05).

**Figure 5:**
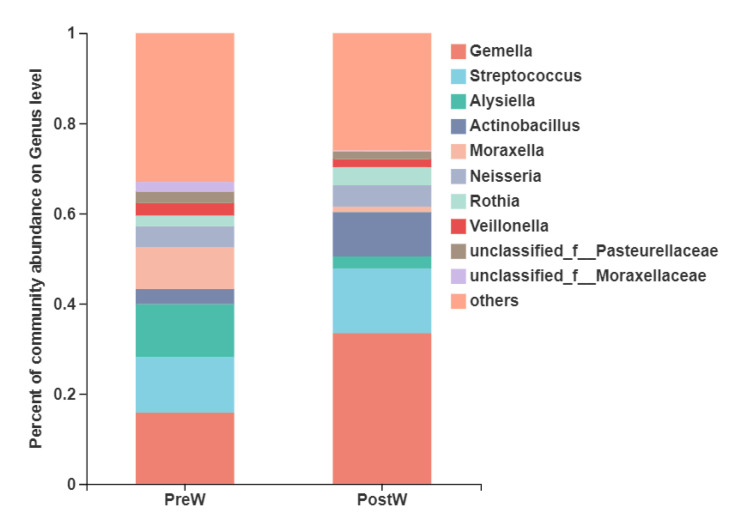
Composition of the predominant oral bacteria at genus level between preweaning and postweaning donkeys (abundance of the genera was expressed as %). PreW, preweaning group; PostW, postweaning group.

At the genus level (Figure 5), the six predominant genera were *Gemella* (15.8~33.5%), *Streptococcus* (12.3~14.3%), *Alysiella* (2.7~11.7%), *Actinobacillus* (3.4~9.9%), *Moraxella* (1.2~9.2%), and *Neisseria* (4.6~4.7%).

**Figure 6:**
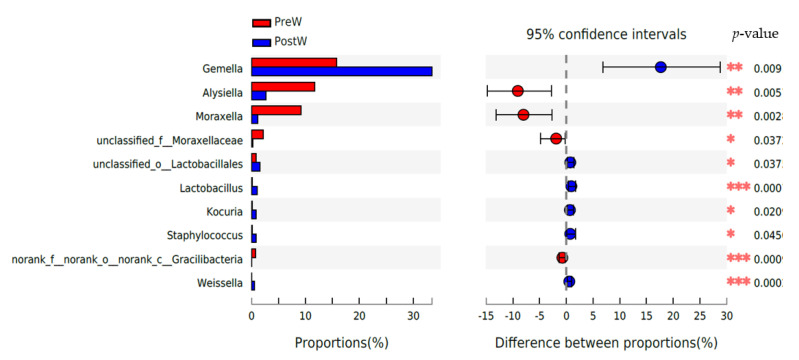
Difference in the predominant oral bacteria at genus level between preweaning and postweaning donkeys (abundance of the genera was expressed as %). Extended error bar plot was created by bioinformatics software (STAMP). Welch’s two-sided test was used and Welch’s inverted was 0.95; PreW, preweaning group; PostW, postweaning group. *, *p* < 0.05; **, *p* < 0.01; ***, *p* < 0.001.

The top 10 differential genera between the two groups were further analyzed (Figure 6). The relative abundance of *Gemella*, unclassified_o__*Lactobacillales*, *Lactobacillus*, *Kocuria*, *Staphylococcus*, and *Weissella* was remarkably greater in the PostW compared with the PreW group (*p <* 0.05). However, the relative abundance of *Alysiella*, *Moraxella*, unclassified_f__*Moraxellaceae*, and norank_f__norank_o__norank_c__*Gracilibacteria* was significantly lower in the PostW than in the PreW group (*p <* 0.05).

### 3.3. LEfSe Analysis of the Oral Microbial Composition

**Figure 7:**
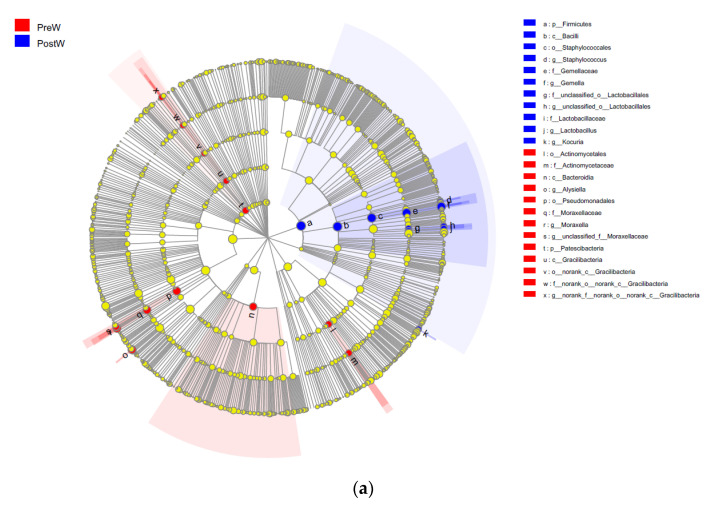

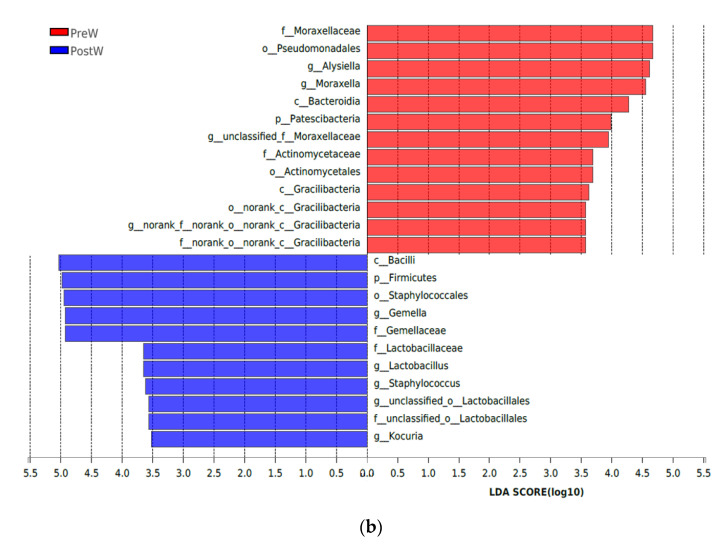
LEfSe analysis of the oral microbial composition of Dezhou donkeys in preweaning and postweaning stages. (**a**) Cladogram by the LEfSe method, visualizing the phylogenetic distribution of the oral bacteria. (**b**) Histogram of the LDA scores, showing the most differentially abundant taxa between two groups (LDA score > 3.5, *n* = 10). PreW, preweaning group; PostW, postweaning group.

A total of 24 biomarkers with statistical differences were determined using LEfSe (8 in the PreW, 11 in the PostW), as shown in Figure 7. *Firmicutes* was significantly enriched in the PostW group, and the PreW group was significantly enriched in the phylum *Patescibacteria*. At genus level, nine genera could be potential biomarkers to distinguish the preweaning and postweaning donkeys. The genus *Alysiella*, *Moraxella*, unclassified_f__*Moraxellaceae*, and norank_f_norank_o_norank_c_*Gracilibacteria* were significantly enriched in the PreW group, whereas the PostW group was significantly enriched by the genera *Gemella*, *Lactobacillus*, and *Staphylococcus*.

### 3.4. Beta Diversity

**Figure 8:**
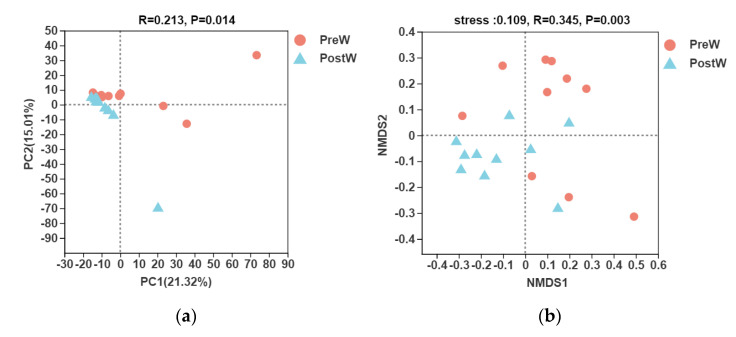
Principal component analysis (PCA, (**a**)) and nonmetric multidimensional scaling analysis (NMDS, (**b**)) of the oral bacteria community composition of the preweaning and postweaning donkeys at the OTU level. The Log10 transformed data were used for analysis, and the percentage values given on each axis represent the amount of total variation. PC1 or MNDS1, first axis; PC2 or MNDS2, second axis. PreW, preweaning group; PostW, postweaning group.

In terms of beta diversity at the OTU level, PCA based on Bray–Curtis dissimilarity showed that the oral microbiota of donkeys was segregated between the PreW and the PostW group (Figure 8a). In addition, the results of the non-metric multidimensional scaling (NMDS, Figure 8b) revealed that there were varieties between individuals for the oral bacteria community composition.

### 3.5. Differences of Oral Bacteria Function between the Two Groups

The microbial functional differences between the two groups were compared. The oral bacteria functions with a relative abundance of ≥1% of the total sequences in at least one of the samples were further analyzed. In total, 32 differential KEGG metabolic pathways (pathway level 3) were detected by PICRUSt (Table S1), and 23 KEGG modules were significantly different between the postweaning and the preweaning donkeys (Table S2).

**Figure 9:**
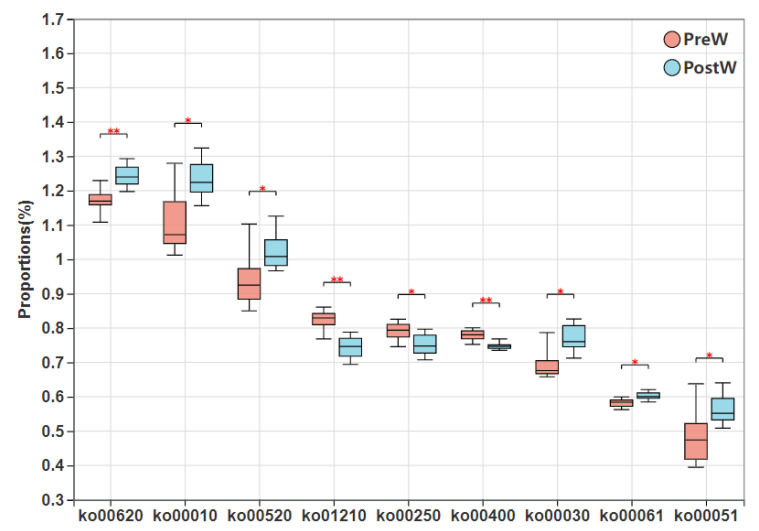
Comparison of enriched KEGG metabolic pathways of preweaning and postweaning donkeys. KEGG, Kyoto Encyclopedia of Genes and Genomes; PreW, preweaning group; PostW, postweaning group; ko00620, pyruvate metabolism; ko00010, glycolysis/gluconeogenesis; ko00520, amino sugar and nucleotide sugar metabolism; ko01210, 2-oxocarboxylic acid metabolism; ko00250, alanine, aspartate, and glutamate metabolism; ko00400, phenylalanine, tyrosine, and tryptophan biosynthesis; ko00030, pentose phosphate pathway; ko00061, fatty acid biosynthesis; ko00051, fructose and mannose metabolism; *, *p* < 0.05; **, *p* < 0.01.

The top seven differential KEGG metabolic pathways between the postweaning and preweaning donkeys are presented in Figure 9. Seventeen of the pathways, including pyruvate metabolism (ko00620), glycolysis/gluconeogenesis (ko00010), amino sugar and nucleotide sugar metabolism (ko00520), pentose phosphate pathway (ko00030), fatty acid biosynthesis (ko00061), and fructose and mannose metabolism (ko00051), were significantly enriched in the PostW group (*p <* 0.05), but 15 differential pathways, such as 2-oxocarboxylic acid metabolism (ko01210), alanine, aspartate and glutamate metabolism (ko00250), and phenylalanine, tyrosine, and tryptophan biosynthesis (ko00400) were higher in the PreW group than in the PostW group (*p <* 0.05).

**Figure 10:**
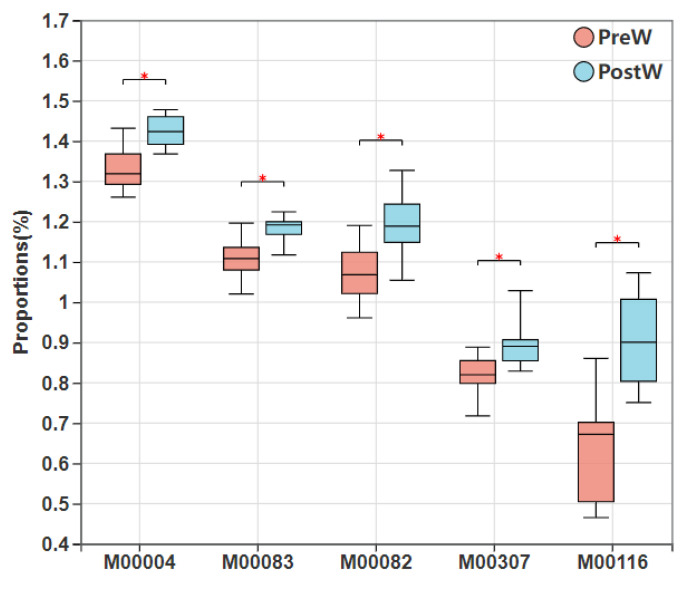
Comparison of enriched KEGG module of preweaning and postweaning donkeys. KEGG, Kyoto Encyclopedia of Genes and Genomes; PreW, preweaning group; PostW, postweaning group; M00004, pentose phosphate pathway (pentose phosphate cycle); M00083, fatty acid biosynthesis (elongation); M00082, fatty acid biosynthesis (initiation); M00307, pyruvate oxidation, pyruvate => acetyl-CoA; M00116, menaquinone biosynthesis, chorismate => menaquinol; *, *p* < 0.05.

Figure 10 shows the top five differential KEGG modules between the PreW and PostW donkeys. There were 14 differential pathways, including pentose phosphate pathway (Pentose phosphate cycle, M00004), fatty acid biosynthesis (elongation, M00083), fatty acid biosynthesis (initiation, M00082), pyruvate oxidation, pyruvate → acetyl-CoA (M00307) and menaquinone biosynthesis, horismite → menaquinol (M00116) significantly enriched in the PreW group (Figure 10, *p <* 0.05).

**Figure 11:**
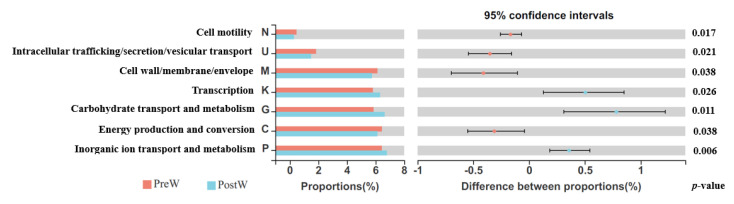
Comparison of COG functional abundance in oral microbiota between preweaning and postweaning donkeys. Proportion of COG abundance differences within the 95% confidence interval. COG, clusters of orthologous groups of proteins; PreW, preweaning group; PostW, postweaning group.

Compared with the COG, a total of 23 s-level classifications were annotated (Figure 11). A total of 11 annotations were significantly different between the PreW and PostW groups, including cell motility, intracellular trafficking/secretion/vesicular transport, cell wall/membrane/envelope biogenesis, transcription, carbohydrate transport and metabolism, energy production and conversion, and inorganic ion transport and metabolism.

## 4. Discussion

Weaning is an important phase of animal production marked by a multitude of biological and environmental stressors, which have a significant impact on the microbiological composition of animals’ digestive tracts [24]. Until now, several studies have been focused on how the digestive tract microbiome develops and changes during weaning periods [15,16,24]. However, information relating to the oral microbiome is limited.

The oral cavity, a part of the digestive system, provides a unique environment for the colonization of microorganisms [12]. In the present study, we compared the bacterial composition of the oral microbiome by 16S rRNA sequencing, and identified bacterial species associated with preweaning and postweaning periods in donkey foals. In order to eliminate the influence of the individual’s sex and environmental condition on the oral microbiota composition in donkeys, all the donkeys in the present study were male and from the same farm. In the current study, the Simpson index of the oral bacteria in the postweaning donkeys was higher than that of the preweaning donkeys, which suggested that the cessation of breastfeeding and weaning onto solids might increase the oral microbiota diversity in donkey foals. This is in agreement with the findings of Amin et al. [16], who reported that the oral microbial diversity increased significantly from the pre- to the post-weaned state in calves. However, a limitation of this study was that the samples were obtained from different animals in the PreW and PostW groups, which might have resulted in variability derived from the different individuals. Although the genetic kinship, geographical environment, and feeding management of donkey foals were similar, further study is needed to monitor the shift in oral microbes in the same individuals.

The predominant phyla in the donkeys’ oral cavities were *Firmicutes*, *Proteobacteria*, *Actinobacteriota*, *Bacteroidota*, *Fusobacteriota*, and *Patescibacteria*. This is in agreement with the findings of Zhu et al. [18], who investigated the influence of dental care on the oral microbiomes of donkeys. In addition, the oral bacteria in the donkey shared some similarities with those of humans and other animals [25,26,27,28,29]. These predominant bacteria at the phylum level were commonly found in the mouths of healthy humans [30], canines [31], and equines [32]. However, the oral microbiome may show large and rapid changes in activity and composition with the response to the shifts in diet [33]. Studies in humans have shown that an obvious increase in *Firmicutes*-dominant bacteria accompanies the introduction of solid foods, including indigestible carbohydrates, into an infant’s diet [34,35]. During the weaning period, donkeys are exposed to a number of stressors, among the most important being the abrupt change in diet from milk to plant-based solid feed. Therefore, the weaning caused the changes in oral bacteria composition in the present study. The relative abundance of oral *Firmicutes* and *Myxococcota* in donkeys was significantly increased after weaning. However, the relative abundance of *Patescibacteria* and *Spirochaetota* was decreased in the postweaning donkeys. Until now, oral microbiome alterations have mainly been associated with dental disease in donkeys, but no information has been reported on the donkey oral microbiome during the weaning period. To the best of our knowledge, the present study is the first investigation comparing the microbial changes before and after weaning in donkeys. It is presumable that the major changes observed in the oral microbiota composition were mainly caused by weaning related dietary shifts. Considering that the donkey oral microbiota profiles significantly differed between the preweaning and postweaning donkeys as diet changes during the weaning period, the nutritional adaptations of the microbial profile may be beneficial in shaping the composition and function of oral microbiota.

At the genus level, the predominant genera detected in the present study were *Gemella*, *Streptococcus*, *Alysiella*, etc. Zhu et al. [18] also reported that the *Gemella* spp. and *Streptococcus* spp. were the most common bacterial genera in the donkey oral cavity. However, weaning had a significant influence on the bacterial genera. The *Gemella* and *Lactobacillales* was more prominent in the postweaning donkeys, highlighting compositional changes within the oral microbiota during the weaning period. *Gemella* has been shown to be more predominant in healthy horses [32]. Hurley et al. [23] noticed that the increases in the genera *Gemella* in the human oral cavity coincided with the eruption of the deciduous teeth and introduction of solid foods to the infant. Aas et al. [36] also found changes in the oral microbiome with the eruption of teeth and in the infant gut microbiota after the administration of solid foods. Consistent with the findings from the human oral microbiome, the relative abundance of oral *Gemella* in donkeys was also increased with the change in diet from milk to plant-based solid feed after weaning. The oral *Lactobacilli* have antimicrobial properties and probiotic qualities [37]. However, oral *Lactobacilli* ware also members of a group of bacteria implicated in caries progression [38]. Until now, the information regarding oral *Lactobacilli* transmission and colonization in donkeys is not well established. However, the oral microbiota of donkey foals undergoes a rapid ecological succession upon the induction of diet changes during the weaning period. The weaning period, which involves diet changes from breast milk to plant-based feeds, may promote the abundance of donkey oral *Lactobacilli*. By contrast, the relative abundance of the genera *Alysiella* and *Moraxella* was greater in the preweaning donkeys in comparison with the postweaning donkeys. *Alysiella* is considered a common resident in the oral cavities of many animals, and it is apparently nonpathogenic [39]. *Moraxella* species have been isolated from a variety of mammalian hosts, including humans, pigs, rabbits, and sheep [40]. They are generally considered to be part of the normal flora on mucosal surfaces [40]. So far, little information has been obtained about the *Alysiella* and *Moraxella* genii during the weaning period in oral microbiome studies. Further studies are needed to determine whether *Alysiella* and *Moraxella* are associated with feed changes during the weaning transition in donkeys.

The oral microbiome is important for maintaining oral homeostasis and protecting the oral cavity [13]. However, no previous studies characterized the microbial function of the oral microbiome in donkeys. In humans, oral microbiota communities have been found to play an important role in physiological, metabolic, and immunological functions, which include the digestion of food and nutrition, the generation of energy, the processing of environmental chemicals, and the maintenance of the immune system [12,41]. In the present study, PICRUSt was applied to observe the metabolic functional changes in oral bacteria between preweaning and postweaning donkeys. The microbial gene functions, including 2-oxocarboxylic acid metabolism, alanine, aspartate, and glutamate metabolism, and phenylalanine, tyrosine, and tryptophan biosynthesis, were enriched in the preweaning donkey foals, and were related to the control of amino acid utilization and metabolic regulation. However, the functions linked with carbohydrate metabolic pathways, such as pyruvate metabolism, glycolysis/gluconeogenesis, amino sugar and nucleotide sugar metabolism, pentose phosphate pathway, and fatty acid biosynthesis, were significantly enriched in the oral microbiome in the postweaning donkeys. These results suggested that the functional repertoire of donkey oral microbiota changes during the weaning period, as the early microbiota before weaning are enriched in microbiota with genes that facilitate lactate utilization, whereas, after weaning, plant-based feeds promote the growth of bacteria enriched in genes coding to allow the utilization of a larger variety of carbohydrates, xenobiotic degradation, and vitamin synthesis. However, additional studies are required to further determine the microbial functions of oral bacteria in donkeys.

## 5. Conclusions

The present study is the first investigation of the donkey oral microbiome in association with weaning. Compared to the preweaning donkeys, the cessation of breastfeeding and weaning onto solids increased the oral bacteria diversity in the postweaning donkeys, with a higher Simpson index. We observed changes in the composition of the oral microbiota in the preweaning and postweaning donkey foals. At the phylum level, the relative abundance of *Firmicutes* and *Myxococcota* was significantly greater in the postweaning donkeys than in the preweaning donkeys. At the genus level, the *Gemella*, unclassified_o__*Lactobacillales*, and *Lactobacillus* were increased in the postweaning donkeys. In addition, the functions related to the carbohydrate metabolic pathways were remarkably enriched in the oral microbiome in the postweaning donkeys. Our findings provide a deeper insight into the oral microbiota changes during the weaning period, as well as the effects of weaning. Together with the documented alterations in diversity and composition, this will help us to obtain a better understanding of the long-term health impact within the oral cavities of donkey foals. However, a limitation of the present study was the variability derived from different individuals, as the samples were obtained from different animals in the PreW and PostW donkeys. Further studies are required in the same individuals to determine which bacteria are actively involved and to confirm how weaning transition can affect the oral health outcomes and oral microbial functions of donkey foals.

## Data Availability

The 16S rRNA gene sequence data in the present study were deposited in NCBI Sequence Read Archive (SRA) under accession number PRJNA851537.

## Supplementary Materials

The following supporting information can be downloaded at: https://www.mdpi.com/article/10.3390/ani12162024/s1. Table S1: Differential KEGG metabolic pathways (pathway level 3) between postweaning and preweaning donkeys predicted by PICRUSt; Table S2: Differential KEGG modules between postweaning and preweaning donkeys predicted by PICRUSt.
