# Supplementary material for: Could Weaning Remodel the Oral Microbiota Composition in Donkeys? An Exploratory Study
Source: Animals (Basel). 2022 Aug 10;12(16):2024. doi: 10.3390/ani12162024 (PMC9404433; doi:10.3390/ani12162024)
Supplement: Supplementary file 1 [file animals-12-02024-s001.zip › animals-1821310-supplementary.pdf]

animals MDPI

Table S1. Differential KEGG metabolic pathways (pathway level 3) between postweaning and preweaning donkeys predicted by PICRUSt

Name	Description	PreW (%)	PostW (%)	Fold Change (PostW/PreW)	*p*-value
ko00010	Glycolysis / Gluconeogenesis	1.11±0.09	1.24±0.06	1.12	0.030
ko00620	Pyruvate metabolism	1.17±0.03	1.24±0.03	1.06	0.006
ko00520	Amino sugar and nucleotide sugar metabolism	0.94±0.07	1.02±0.05	1.09	0.042
ko00030	Pentose phosphate pathway	0.70±0.04	0.77±0.04	1.11	0.028
ko00250	Alanine, aspartate and glutamate metabolism	0.79±0.03	0.75±0.03	0.95	0.048
ko00400	Phenylalanine, tyrosine and tryptophan biosynthesis	0.78±0.02	0.75±0.01	0.96	0.010
ko01210	2-Oxocarboxylic acid metabolism	0.82±0.03	0.74±0.03	0.90	0.008
ko00061	Fatty acid biosynthesis	0.58±0.01	0.60±0.01	1.04	0.023
ko00051	Fructose and mannose metabolism	0.48±0.07	0.56±0.04	1.17	0.042
ko00330	Arginine and proline metabolism	0.42±0.03	0.48±0.05	1.13	0.042
ko01501	beta-Lactam resistance	0.47±0.05	0.43±0.03	0.93	0.048
ko00670	One carbon pool by folate	0.46±0.01	0.43±0.02	0.93	0.018
ko04112	Cell cycle - Caulobacter	0.46±0.02	0.43±0.01	0.95	0.030
ko00220	Arginine biosynthesis	0.46±0.02	0.42±0.03	0.92	0.037
ko00561	Glycerolipid metabolism	0.35±0.05	0.41±0.04	1.18	0.037
ko00760	Nicotinate and nicotinamide metabolism	0.46±0.03	0.41±0.04	0.88	0.036
ko00910	Nitrogen metabolism	0.41±0.04	0.36±0.03	0.88	0.042
ko04066	HIF-1 signaling pathway	0.27±0.03	0.31±0.02	1.15	0.036
ko04922	Glucagon signaling pathway	0.24±0.03	0.28±0.02	1.18	0.030
ko05230	Central carbon metabolism in cancer	0.21±0.03	0.26±0.01	1.24	0.021
ko01523	Antifolate resistance	0.19±0.01	0.18±0.01	0.94	0.048
ko00473	D-Alanine metabolism	0.13±0.02	0.17±0.03	1.24	0.048
ko00750	Vitamin B6 metabolism	0.19±0.01	0.17±0.01	0.90	0.037

ko04213	Longevity regulating pathway - multiple species	0.13±0.01	0.14±0.01	1.13	0.028
ko05150	Staphylococcus aureus infection	0.07±0.04	0.13±0.04	1.82	0.030
ko00471	D-Glutamine and D-glutamate metabolism	0.14±0.01	0.13±0.01	0.95	0.037
ko00625	Chloroalkane and chloroalkene degradation	0.09±0.02	0.12±0.01	1.34	0.037
ko05418	Fluid shear stress and atherosclerosis	0.14±0.01	0.12±0.01	0.84	0.030
ko00785	Lipoic acid metabolism	0.09±0.01	0.11±0.01	1.16	0.023
ko02025	Biofilm formation - Pseudomonas aeruginosa	0.14±0.02	0.11±0.01	0.81	0.048
ko05014	Amyotrophic lateral sclerosis (ALS)	0.08±0.01	0.10±0.01	1.20	0.030
ko04626	Plant-pathogen interaction	0.12±0.01	0.10±0.01	0.83	0.023

PreW, preweaning group; PostW, postweaning group.

Table S2. Differential KEGG modules between postweaning and preweaning donkeys predicted by PICRUSt

Name	Description	PreW(%)	PostW(%)	Fold Change (PostW/PreW)	*p*-value
M00004	Pentose phosphate pathway (Pentose phosphate cycle)	1.33±0.05	1.42±0.04	1.07	0.023
M00006	Pentose phosphate pathway, oxidative phase, glucose 6P => ribulose 5P	1.07±0.08	1.19±0.09	1.12	0.044
M00025	Tyrosine biosynthesis, chorismate => tyrosine	1.11±0.05	1.18±0.03	1.06	0.041
M00028	Ornithine biosynthesis, glutamate => ornithine	0.64±0.15	0.91±0.12	1.41	0.027
M00035	Methionine degradation	0.82±0.05	0.89±0.06	1.09	0.044
M00045	Histidine degradation, histidine => N-formiminoglutamate => glutamate	0.54±0.11	0.72±0.11	1.32	0.028
M00082	Fatty acid biosynthesis, initiation	0.59±0.04	0.52±0.05	0.88	0.028
M00083	Fatty acid biosynthesis, elongation	0.30±0.13	0.50±0.13	1.69	0.048
M00093	Phosphatidylethanolamine (PE) biosynthesis, PA => PS => PE	1.09±0.37	0.47±0.27	0.43	0.037
M00116	Menaquinone biosynthesis, chorismate => menaquinol	0.35±0.05	0.44±0.02	1.24	0.012
M00124	Pyridoxal biosynthesis, erythrose-4P => pyridoxal-5P	0.24±0.13	0.42±0.14	1.77	0.048
M00144	NADH:quinone oxidoreductase, prokaryotes	0.64±0.09	0.42±0.13	0.66	0.028
M00155	Cytochrome c oxidase, prokaryotes	0.41±0.03	0.39±0.01	0.95	0.044
M00307	Pyruvate oxidation, pyruvate => acetyl-CoA	0.22±0.08	0.36±0.08	1.61	0.044
M00367	C10-C20 isoprenoid biosynthesis, non-plant eukaryotes	0.39±0.05	0.30±0.04	0.78	0.023
M00580	Pentose phosphate pathway, archaea, fructose 6P => ribose 5P	0.13±0.08	0.29±0.11	2.15	0.031
M00569	Catechol meta-cleavage, catechol => acetyl-CoA / 4-methylcatechol => propanoyl-CoA	0.19±0.06	0.29±0.07	1.50	0.048
M00416	Cytochrome aa3-600 menaquinol oxidase	0.37±0.06	0.29±0.04	0.79	0.048
M00609	Cysteine biosynthesis, methionine => cysteine	0.17±0.06	0.28±0.05	1.61	0.028
M00725	Cationic antimicrobial peptide (CAMP) resistance, dltABCD operon	0.25±0.02	0.22±0.02	0.87	0.041
M00842	Tetrahydrobiopterin biosynthesis, GTP => BH4	0.25±0.02	0.22±0.02	0.87	0.041
M00843	L-threo-Tetrahydrobiopterin biosynthesis, GTP => L-threo-BH4	0.38±0.13	0.21±0.09	0.55	0.041
M00845	Arginine biosynthesis, glutamate => acetylcitrulline => arginine	0.08±0.03	0.13±0.02	1.56	0.044

PreW, preweaning group; PostW, postweaning group.
